# Characterization of an isobutylene epoxide hydrolase (IbcK) from the isobutylene-catabolizing bacterium *Mycolicibacterium* sp. ELW1

**DOI:** 10.1128/aem.00393-25

**Published:** 2025-08-26

**Authors:** Nicholas W. Faulkner, John B. Joyce, Christy Smith, Paul Swartz, Robert B. Rose, Eric S. Miller, Michael R. Hyman

**Affiliations:** 1 Department of Plant and Microbial Biology, North Carolina State University 6798 , Raleigh, North Carolina, USA; 2 Department of Molecular and Structural Biochemistry, North Carolina State University 6798 , Raleigh, North Carolina, USA; Universidad de los Andes, Bogotá, Colombia

**Keywords:** epoxide hydrolase, isobutylene, isobutylene oxide, MTBE, alkenes, bioremediation

## Abstract

Isobutylene (IB) is produced on a large scale by the petrochemical industry and is metabolized by the aerobic alkene-metabolizing bacterium *Mycolicibacterium* sp. ELW1. The initial metabolite of IB catabolism by this bacterium is proposed to be 2-methyl-1,2-epoxypropane (isobutylene oxide [IBO]). The epoxide is then thought to be rapidly converted into 2-methyl-1,2-propanediol (MPD) by an epoxide hydrolase. A gene (*ibcK*) encoding a hydrolase is in a putative IB catabolism gene cluster on a ~222-kbp megaplasmid. This gene was cloned, heterologously expressed, and purified by Ni-NTA affinity chromatography. The purified protein rapidly and stoichiometrically hydrolyzed IBO to MPD with a specific activity of 29 µmoles min^−1^ mg protein^−1^. Additional epoxides were also hydrolyzed by IbcK, including 1,2-epoxypropane, 1,2-epoxybutane, 1,2-epoxypentane, epichlorohydrin, and cyclohexane oxide, albeit at lower rates than IBO. IbcK also slowly hydrolyzed both *cis*- and *trans*-2,3-epoxybutane, which are the only other epoxides other than IBO known to support the growth of *Mycolicibacterium* sp. ELW1. Furthermore, IbcK also appears to be enantioselective towards chiral *trans* 2,3-epoxybutane. The crystal structure of IbcK was determined at 2.29 Å resolution, revealing a two-domain structure with an α/β hydrolase fold topology at its core. IbcK has high similarity to the epoxide hydrolase EchA from *Agrobacterium radiobacter* AD1, including the key active site residues Asp 117, Asp 256, and His 284. IbcK was observed to be in monomer–dimer equilibrium, which we propose occurs through interactions between the “cap” domains.

**IMPORTANCE:**

The initial metabolites generated during catabolism of volatile alkenes by aerobic alkene-oxidizing bacteria are consistently epoxides. These bacteria employ several different mechanisms to protect DNA, lipids, and proteins from damage by these reactive metabolites. The most common mechanisms are conjugation with coenzyme M or glutathione. In contrast, the role for hydrolases in the bacterial metabolism of volatile alkenes and their epoxides has not been frequently observed. The enzymatic, functional, and structural characterization of an epoxide hydrolase (IbcK) from the IB-utilizing bacterium *Mycolicibacterium* sp. ELW1 described here advances our understanding of these enzymes and suggests their potential application as an enantioselective catalyst. This study advances our understanding of how microorganisms utilize aliphatic alkenes, such as carbon and energy sources, including the role of epoxide hydrolases in these catabolic pathways.

## INTRODUCTION

Aerobic bacterial growth on volatile alkenes, such as ethene, propene, and isoprene (2-methyl-1,3-butadiene), has been extensively characterized, and much is known about the microorganisms, pathways, and enzymes involved in the catabolism of these compounds (1–5). The catabolism of alkenes by aerobic alkene-oxidizing bacteria typically involves the initial oxidation of the C = C bond by monooxygenases, leading to the production of epoxides. In contrast, the enzymes and mechanisms involved in the further metabolism of these epoxides are more diverse. For example, the catabolism of 1,2-epoxypropane by propene-grown *Xanthobacter autotrophicus* Py2 involves initial epoxide conjugation with coenzyme-M (CoM) (6, 7), and the same CoM-dependent process also occurs in bacteria that can grow on C_2_ alkenes, such as ethene and chloroethene (8). In contrast, the epoxide generated from the initial monooxygenase-catalyzed oxidation of isoprene is further metabolized after conjugation with glutathione (GSH) (4, 9).

While the role of epoxide hydrolases in lipid metabolism and signaling molecule transformations has been well studied in eukaryotes (10, 11), these enzymes have been less frequently studied in bacterial systems and are certainly unusual in bacterial alkene catabolism. EchA from *Agrobacterium radiobacter* AD1 is perhaps the best characterized bacterial epoxide hydrolase and is responsible for initiating aerobic epichlorohydrin (1-chloro-2,3-epoxypropane) catabolism by hydrolyzing this epoxide to 3-chloro-2,3-propanediol (12). Limonene epoxide hydrolase also plays a similar role in terpene catabolism by *Rhodococcus erythropolis* DCL14 (13). The diverse epoxide hydrolases in *Mycobacterium tuberculosis* have also been studied as potential therapeutic targets due to their suspected roles in lipid metabolism and detoxification processes in macrophage hosts (14, 15).

 In the present study, we have focused on the putative role of an epoxide hydrolase in the catabolism of 2-methylpropene (isobutylene) by *Mycolicibacterium* sp. ELW1 (formerly *Mycobacterium* sp. ELW1), hereafter referred to as ELW1. Isobutylene (IB) is one of the four butene isomers and is the simplest branched alkene. This gas is produced on a large scale by the petrochemical industry and is primarily used as a feedstock for the synthesis of high-volume products, including butyl rubber, isooctane, and gasoline oxygenates, such as methyl *tertiary*-butyl ether (MTBE) (16). ELW1 was originally isolated from aerobic enrichment cultures supplied with IB as the sole carbon and energy source (17). This bacterium grows rapidly (0.05 h^−1^) on IB, compared to growth on *cis*- and *trans*- 2-butene, which are the only other identified alkene growth substrates for this bacterium (17). Although neither isoprene nor C_2_–C_4_ *n*-alkenes support growth of this strain (17), IB-grown cells of strain ELW1 can co-oxidize several gaseous *n*-alkenes (18). In the case of ethene, this leads to the accumulation of 1,2-epoxyethane, whereas 1,2-epoxypropane, 1,2-epoxybutane, and 2-methyl-1,2-epoxybutane do not accumulate during oxidation of their C_3_–C_5_ alkenes as they are all rapidly consumed by IB-grown cells (18). These observations suggest that, like other gaseous alkene-metabolizing strains, the initial reaction in the pathway of IB catabolism in ELW1 involves a monooxygenase-catalyzed oxidation of the C = C bond to generate 1,2-epoxy-2-methylpropane (IBO).

**Fig 1:**
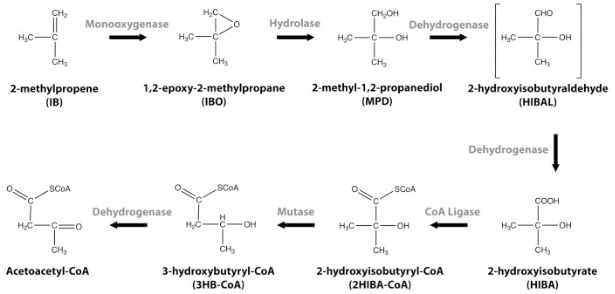
Proposed pathway for isobutylene (IB) catabolism in *Mycolicibacterium* sp. ELW1. The IbcK epoxide hydrolase catalyzes the second reaction, converting IBO to MPD.

Physiological studies suggest that epoxide conjugation with CoM does not occur in ELW1, as IBO is a potent mechanism-based inactivator of the key enzyme responsible for conjugating CoM with epoxides (7). The oxidation of IB by ELW1 is also not inhibited by 2-bromoethanesulfonate (BES), another potent inhibitor of CoM-dependent epoxide conjugation (19). In contrast, our prior studies suggest that IBO is enzymatically hydrated to 2-methyl-1,2-propanediol (MPD) (17). The proposed pathway of IB catabolism in strain ELW1 is shown in Fig. 1, with the second step represented as an epoxide hydrolase-catalyzed reaction.

The complete genome sequence of ELW1 was obtained through NCBI, and an epoxide hydrolase CDS (GenBank locus QEN17571.1) was identified in a cluster of plasmid-borne genes that also encode a putative Group 2 soluble di-iron monooxygenase (SDIMO). This monooxygenase is strikingly similar to alkene monooxygenases found in the model propene-oxidizing strain *X. autotrophicus* Py2 (20), the model isoprene-oxidizing bacterium *Rhodococcus* sp. strain AD45 (21), and two newly characterized IB-metabolizing bacteria, *Mycolicibacterium gadium* IBE100 and *Mycobacterium paragordonae* IBE200 (22). Further evidence supporting the proposed pathway and function of this epoxide hydrolase was provided by a genetic comparison to *M. gadium* IBE100 and *M. paragordonae* IBE200 (22). Each of these species possesses IB-catabolizing (*ibc*) gene clusters strikingly similar to ELW1, with *M. gadium* IBE100 having 100% amino acid similarity between IbcK and its corresponding epoxide hydrolase (22). Transcriptomic data obtained for *M. paragordonae* IBE200 grown on IB revealed significant upregulation of all the *ibc* genes, including the orthologous *ibcK* for epoxide hydrolase (22). Additionally, the orthologous IbcK was identified by peptide-mass fingerprinting from *M. gadium* IBE100 when grown on IB (22). In this study, we describe the expression, purification, activities, and crystal structure of the heterologously expressed IbcK (isobutylene catabolism gene K product) from *Mycolicibacterium* sp. ELW1. Our results indicate that this protein functions *in vivo* as an epoxide hydrolase and provide insights into important structural and catalytic features of the enzyme and its likely role in IB catabolism.

## RESULTS AND DISCUSSION

### Identification of the putative epoxide hydrolase IbcK

The complete genome of *Mycolicibacterium* sp. ELW1 (accessions CP032155.1 [chromosome] and CP032156.1 [plasmid]) contains genes located on a plasmid that encode a putative alkene monooxygenase and a 303 amino acid protein with a predicted mass of ~34,626 Da. BLASTP analysis of this putative enzyme yielded six aligned proteins with *S-*score > 200. One of these, EchA, was from the epichlorohydrin-utilizing bacterium, *A. radiobacter* sp. AD1. The genome of ELW1 contains three additional putative epoxide hydrolases encoded on its chromosome. A phylogenetic tree based on the predicted amino acid sequences of these proteins and other known bacterial epoxide hydrolases revealed that the plasmid-borne epoxide hydrolase from ELW1 is closely related to the epoxide hydrolases expressed by *M. gadium* IBE100 and *M. paragordonae* IBE200 during growth on IB (22) (Fig. S1). The identical amino acid identity of IbcK to its homolog in *M. gadium* IBE100 likely reflects a similar function and substrate range for these two enzymes. Although enzymatic assays have not been reported for the epoxide hydrolases from *M. gadium* IBE100 or *M. paragordonae* IBE200 (22), the expression data and sequence similarities strongly suggest an active role of the orthologous IbcK enzymes in IB catabolism. In contrast, the other putative epoxide hydrolases encoded on the ELW1 chromosome are only distantly related to the plasmid-encoded enzyme from ELW1 and those from *M. gadium* IBE100 and *M. paragordonae* IBE200.

### Expression and purification of IbcK

The *ibcK* gene encoding a putative epoxide hydrolase was cloned into pET28a(+) (a T7 promoter vector) that adds six histidine residues and a thrombin cleavage site (20 amino acids total) to the *N*-terminus of the protein (Fig. S2). The plasmid was transformed into *E. coli* BL21(DE3), and expression was induced with IPTG, as described in Materials and Methods. After cell disruption, the expressed protein was purified using Ni-NTA affinity binding and elution. These steps resulted in a 15.4-fold purification of IbcK with a yield of ~15% (Table S1). After the Ni-NTA affinity purification step, the measured specific activity of the purified enzyme with IBO as a substrate was 29 µmoles MPD produced min^−1^ mg total protein^−1^, with a total of 601 units recovered.

SDS-PAGE analysis of increasing amounts (2.5–20 µg) of purified IbcK (Fig. S3a) indicated the presence of minor polypeptides with lower masses than expected for the purified protein (Fig. S3b). These polypeptides were not removed using size exclusion chromatography and subsequent dialysis. These attempts at further purification also slightly decreased both the yield and specific activity of the final protein preparation (Table S1). These minor proteins were excised from an SDS-PAGE gel and subjected to in-gel tryptic digestion. An LC/MS analysis of the resulting tryptic fragments indicated that the lower molecular weight contaminating proteins were degradation products of IbcK (data not shown).

### Transformation of isobutylene oxide (IBO) by IbcK

**Fig 2:**
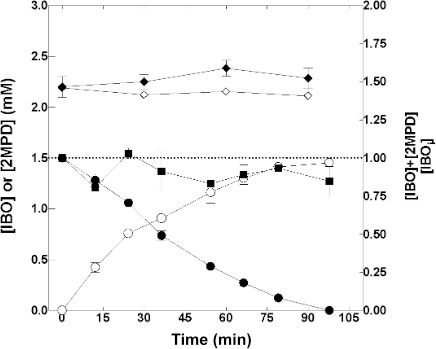
Stoichiometry of conversion of IBO to MPD by recombinant IbcK. Purified IbcK was incubated with IBO ([initial] = 1.5 mM), and the time course of its conversion to MPD was determined by periodic analyses of the reaction mixture by GC. The lower panel shows the corresponding changes in the concentrations of IBO (●) and MPD (○) and the sum of both [IBO] and [MPD] (◼) at each time point in reactions containing IbcK (3 µg). The upper panel shows the changes in IBO (⧫) and MPD (◊) concentration ([initial] = 2.25 mM) over time in abiotic reactions conducted without IbcK. The incubation conditions and GC analyses were conducted as described in Materials and Methods. The data presented are the means and range of two technical replicates.

Prior physiological studies suggest that the initial metabolite in IB catabolism by ELW1 is IBO (17). For example, IB-grown cells rapidly hydrolyze IBO to MPD (17), and with high initial IBO concentrations (5 mM), the mass balance of this reaction reaches a maximum of ~1:1 for IBO consumed relative to MPD produced. To explore the likely physiological role of IbcK in IBO hydrolysis, the time course of IBO transformation by purified IbcK was therefore examined. Both rapid consumption of IBO and accumulation of MPD were simultaneously initiated when IbcK (3 µg) was added to the reaction mixture (Fig. 2). The enzyme remained active throughout the 100-min reaction time course, and MPD was the only reaction product detected by the analytical approaches used in this study. The sum of the dissolved concentrations of IBO and MPD relative to the starting concentration of IBO in the reaction was consistently in the range of 0.78 to 1.06 (mean = 0.91 [*n* = 8]) (Fig. 2). No transformation of either IBO or MPD was observed over slightly shorter (90 min) abiotic control incubations conducted without IbcK and higher initial concentrations (2.25 mM) of these compounds (Fig. 2). Based on these observations, we conclude that IbcK catalyzes the stoichiometric conversion of IBO to MPD. The results shown in Fig. 2 also indicate that the rate of IBO transformation was close to constant over the first 45 min of the reaction, whereas at the latest time points (>75 min), the rate decreased over 2-fold, suggesting that the *K*_*m*_ for IBO is ≤200 µM. This estimated *K*_*m*_ value for IBO and the assumed *V*_max_ for IBO hydrolysis by IbcK (29 µmoles min^−1^ mg protein^−1^) are both similar to the corresponding values (~300 µM and 34 µmoles min^−1^ mg protein^−1^) for epichlorohydrin hydrolysis by EchA from epichlorohydrin-grown *A. radiobacter* (formerly *Pseudomonas* sp.) strain AD1 (23).

### Substrate range of IbcK

Using a fixed 2 mM initial epoxide concentration, 3 µg of purified IbcK, and an incubation time of 30 min, IbcK also hydrolyzed other epoxides, albeit much less effectively compared to IBO. The specific activity (µmols/min^-1^/mg protein^-1^ ± s.d.) of IbcK was 17.0 ± 0.4 on IBO, 6.3 ± 0.2 on 1,2-epoxypropane, 4.9 ± 0.8 on 1,2-epoxybutane, 3.8 ± 0.1 on 1,2-epoxypentane, 4.4 ± 1.5 on epichlorohydrin, and 1.0 ± 0.9 on cyclohexane oxide. The specific activity of IbcK for cyclohexane oxide was low, but measurable above the observed loss (≤2%) of these epoxides in abiotic reactions that lacked IbcK (data not shown). Under these experimental conditions, the transformation of *cis-* and *trans*-2,3-epoxybutane bordered the resolution limit for GC analysis and so was evaluated at higher enzyme concentrations described below. Collectively, these data show that IBO is the preferred substrate for IbcK and that the hydrolysis of IBO to MPD is the physiological role of this enzyme.

**Fig 3:**
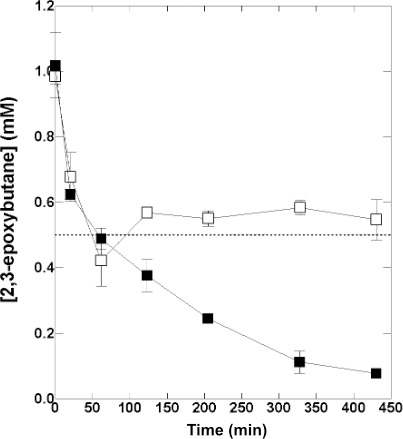
Transformation of *cis*- and *trans*-2-3-epoxybutane by IbcK. The figure shows the time course of the transformation of 1 mM E-C2B (■) and E-T2B (◻) by IbcK (25 µg). The incubation conditions and GC analyses were as described in Materials and Methods. The data presented show the mean and range of two technical replicates.

The only other volatile alkene apart from IB that supports growth of ELW1 is 2-butene; relative to growth on IB, ELW1 grows slowly on both the *cis* and *trans* isomers of this gas (17). ELW1 can also grow on both *cis-* and *trans*-2,3-epoxybutane (E-C2B and E-T2B, respectively) as well as the corresponding butane-2,3-diols (17). However, purified IbcK transformed both epoxides even less efficiently than cyclohexane oxide. In longer-term incubations with increased amounts of IbcK, the time course of E-C2B and E-T2B transformations was indistinguishable over the initial 30 min. However, while transformation of E-C2B continued throughout the reaction time course, transformation of E-T2B effectively ceased after ~60 min. After 450 min when ~90% of the initial E-C2B had been transformed, ~55% of the initial E-T2B still remained in the reaction mixture (Fig. 3). Although the cause of this difference in transformation patterns was not further explored, E-C2B is achiral, whereas E-T2B is chiral and is commercially supplied as a racemic mixture of its two enantiomers. Our results (Fig. 3) suggest that IbcK is enantioselective and preferentially hydrolyzes one of the two E-T2B enantiomers (2R,3S and 2S,3R). While epoxide hydrolases are well known as enantioselective catalysts (24), further studies will be required to confirm this and identify the preferentially transformed E-T2B enantiomer. The structure of IbcK provides some insights into substrate selectivity.

### Structure of IbcK

Diffraction-quality crystals of IbcK were obtained in the presence of 1.6 M MgSO_4_ and 0.1 M MES:NaOH, pH 6.5. The crystals diffracted to 2.29 Å resolution in space group P2_1_2_1_2_1_ (Table S2) with six monomers in the asymmetric unit (Fig. S4). The monomers are identical, superimposing with a root-mean-square error (RMSD) between monomers of 0.19 and 0.25 Å between C-alphas. The final model included 292–294 protein residues per monomer. The first 9–11 residues were disordered and included the 6×His tag and thrombin cleavage site. Also included are 276 water molecules, plus two Mg^2+^ and two SO_4_^2-^ molecules from the crystallization solution. Each monomer also contains one glycerol molecule, used as a cryoprotectant, in the active site, although the glycerol in chain F is not well ordered (see Materials and Methods).

**Fig 4:**
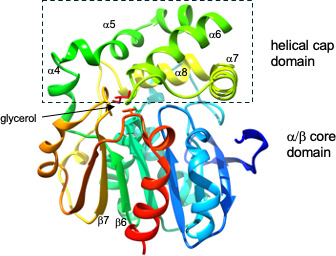
The classical α/β hydrolase fold of the IbcK monomer structure (chain A) colored from blue to red, N- to C-terminus. The α/β core and helical cap domains are labeled, with the cap domain (α4–α8 between β6 and β7) demarcated by a dotted box. Also shown is the position of glycerol in the active site situated between the two domains (PDB 9C2G).

The IbcK monomer structure (PDB 9C2G) displays a classic α/β hydrolase fold (Fig. 4) (25). The α/β “core” domain, residues 1–146 and 229–303, is composed of an eight-stranded beta sheet connected by α-helices. The adjacent α-helical “cap” structure, residues 147–228, consists of five α-helices spliced between strands β6 and β7 and oriented perpendicular to the core domain. The monomer superimposes on the previously determined *A. radiobacter* AD1 epoxide hydrolase structure (PDB 1EHY) with an RMSD of 0.875 for 271 C-alpha atoms.

**Fig 5:**
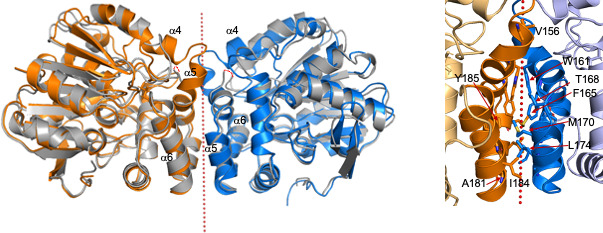
Dimer interface between IbcK chain A (orange) and chain B (blue). (Left) The interface is composed of helices α5 and α6 in the cap domain of both monomers, burying a total of 6,120 Å^2^, half from each monomer (calculated by Areaimol from the CCP4 suite of programs) (29, 30). The 2-fold axis is shown as red dots. The same dimer is found in chains A and C in the AD1 1EHY structure (gray) (31). Two turns of helix α5 (and helix α4) are disordered in the AD1 1EHY structure (red dashed lines represent the missing residues), reducing the apparent interface in the AD1 structure. In the IbcK structure, similar dimers are formed between chains E and F and between chains C and chain D from a different asymmetric unit. (Right) Hydrophobic residues in the dimer interface of IbcK. Residues from only one of the monomers are labeled.

The buried surface area between two monomers in the IbcK structure is 6,120 Å^2^ and suggests a functional dimer (Fig. 5) (26, 27). The two cap domains interact in the dimer, composed of helices α5, α6, and α7, burying eight hydrophobic residues from each monomer, again characteristic of a dimer interface (28). Interestingly, the same dimer potentially exists in the EchA structure. However, the two turns of helix α5 are disordered in the structure, reducing the dimerization interface. EchA was previously reported as a monomer (12).

Formation of a dimer by the purified IbcK was tested in solution by size exclusion chromatography (Fig. S5). The profile contained two peaks at molecular masses of 67,200 and 46,200 Da, compared with the deduced molecular mass of the IbcK monomer of 36,789 Da, including the His tag. The first peak may represent the dimer, and the second peak the monomer, suggesting an equilibrium between monomer and dimer in solution.

### IbcK active site

**Fig 6:**
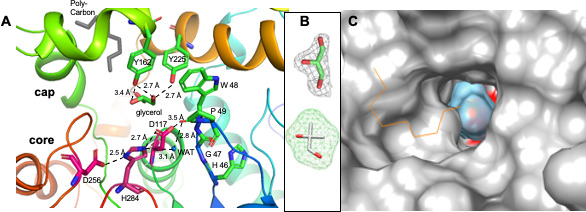
Glycerol bound to the IbcK active site resembles the 2-methyl-1,2-propanediol (MPD) product complex**.** (**A**) The two hydroxyls of glycerol form hydrogen bonds with the side chain hydroxyls of Tyr 162 and Tyr 225. Also shown are the catalytic triad (magenta: D117, D256, H284), the oxyanion hole (H46, G47, W48, P49), and the proposed catalytic water, water 80 (WAT). Distances are indicated by dotted lines. The cartoon representation is colored from blue to red, from N to C, as shown in Fig. 5. Electron density blocking the active site was filled with a poly-carbon chain (gray, labeled as chain K, residue UNL in the PDB file). (**B**) Final 2FoFc density for the glycerol in the active site of chain A (top) compared with a model of MPD (ChemSpider: CSID:61693), the product of the IbcK reaction, with a surface drawn as a mesh. (**C**) Surface rendering of IbcK (gray) demonstrates that glycerol (blue surface) occupies most of the IbcK binding pocket. The active site D117 is located at the base of the binding pocket, behind the glycerol (colored red). The poly-carbon chain is shown as an orange line (images generated in Chimera).

The active site of IbcK is in the cavity between the core and lid domains, as in other α/β hydrolases (Fig. 6). The active site residues described for EchA are conserved in IbcK (Fig. S6) (29). This includes the three active site residues located on loops of the core domain: Asp 117 on the “nucleophile elbow” between β5 and α3, Asp 256 after β7, and His 284 after β8 (AD1 Asp 197, Asp 248, and His 275, respectively) (Fig. 6) (25). In the proposed catalytic mechanism for EchA, Asp 117 attacks a carbon of the epoxide ring forming an ester linkage with the Asp side chain (25). Tyr 162 and Tyr 225 (AD1 Tyr 152/Tyr 215) facilitate ring opening by neutralizing the negative charge or protonating the epoxide oxygen. In the second step, a water molecule attacks the ester carbonyl to regenerate the active site Asp 117. Mutations of the active site His of EchA resulted in single turnover of substrate, consistent with the role of His 284 and Asp 256 in deprotonating the catalytic water (25). This water molecule is identified as water 371, within hydrogen-bonding distance from the active site His (31). Water 80 occupies the same position in the IbcK structure, allowing it to be deprotonated by His 284 (Fig. 6). Backbone nitrogen from the conserved H-G-W-P loop (residues 46–49) after β3 is proposed to form the oxyanion hole (EchA residues 36–39), which includes the W48–P49 *cis* proline (31).

 Electron density in each of the IbcK monomer active sites was built as a glycerol molecule, which was added to the crystals as a cryoprotectant (Fig. 6B). The glycerol is positioned with two hydroxyl groups within hydrogen-bonding distance of Tyr 162 and Tyr 225. As the two hydroxyl groups of glycerol may reflect the IBO hydrolysis product MPD, the two Tyr residues may facilitate product release in IbcK. Glycerol occupies most of the active site cavity, as demonstrated by a surface rendering of IbcK (Fig. 6C). The confined binding site may explain the reduced activity of epoxide substrates by size from 1,2-epoxypropane to 1,2-epoxybutane to 1,2-epoxypentane to C6 as described previously. Furthermore, the reduced activity of the 2,3-epoxybutane isomers suggests the active site is also inaccessible to non-terminal epoxides.

Of note, access to the active site is blocked in each of the six monomers by an unidentified molecule (Fig. 6; Fig. S8). Efforts to model the density as an alternate conformation of a loop failed. The electron density was modeled as poly-carbon as a placeholder (chain K, residue UNL). The significance of this density is not clear and may be an artifact of crystallization.

The original 1992 paper recognizing the α/β hydrolase fold reported monomeric and dimeric examples (25). The cap domain functions as a dimer interface in several bacterial epoxide α/β hydrolases, although the interfaces differ (Fig. S7). Overall, the cap domains are less conserved than the core domains (Fig. S6). Other dimer interfaces also occur; for example, the *M. tuberculosis* epoxide hydrolase B dimer interface spans the core and cap domains (Fig. S7E).

Overall, the results of this study indicate that IbcK is likely responsible for initiating IBO catabolism in ELW1 and that hydrolysis is therefore another important biochemical route for the aerobic microbial transformation of epoxides generated from gaseous alkenes. While IbcK rapidly hydrolyzes IBO (Fig. 2) and ELW1 can grow on this epoxide (17), other epoxides, such as 1,2-epoxypropane and 1,2-epoxybutane, are also hydrolyzed at substantial rates by IbcK, but these compounds do not support growth (17). Conversely, the slowest hydrolysis rates we observed were for *cis*- and *trans*-2,3-epoxybutane, which both support slow growth of ELW1 (17). The unfavorable kinetics of IbcK-catalyzed hydrolysis of E-T2B and E-C2B very likely contributes to the slow growth rate of ELW1 on both the *cis-* and *trans*-2-butene isomers and their corresponding epoxides relative to growth on IB and IBO (17). However, other factors must account for why other more rapidly hydrolyzed epoxides, such as 1,2-epoxypropane and 1,2-epoxybutane, are not utilized as growth-supporting substrates. In these cases, it is likely due to the limited ability of ELW1 to grow on diols rather than the catalytic features of IbcK. In support of this, our prior studies have shown that ELW1 will grow well on MPD and all of the 2,3-butanediol enantiomers, but not on ethylene glycol, 1,2-propanediol, or 1,2-butanediol (17).

Our crystallographic analysis indicates that the structure of IbcK exhibits considerable similarities to EchA from *A. radiobacter* AD1, and several key amino acids identified in prior studies with EchA also participate in the catalytic mechanism of IbcK. Changes in the enantioselectivity of EchA have been investigated through site-directed mutagenesis of these and other key residues (32–34). Further studies with IbcK using this approach may shed light on the likely enantioselectivity of IbcK towards E-T2B and the possibility that this enzyme is also enantioselective towards other known chiral epoxide substrates for IbcK such as 1,2-epoxyalkanes.

## MATERIALS AND METHODS

### Gases and reagents

2-Methyl-1,2-epoxypropane (99%+) and *trans*-2,3-epoxybutane (97%) were obtained from Alfa Aesar (Ward Hill, MA). 1,2-Epoxybutane (99%+), 1,2-epoxypentane (98%), *cis*-2,3-epoxybutane (97%), epichlorohydrin (99%), styrene oxide (97%), and cyclohexene oxide (98%) were obtained from Sigma Aldrich (Saint Louis, MO). 2-Methylpropene (CP grade) and air, N_2_, and H_2_ for gas chromatography were obtained locally. 2-Methyl-1,2-propanediol was obtained from Oakwood Chemical (Estill, SC). Culture media and IPTG were obtained from Thermo Fisher Scientific (Waltham, MA).

### Bacterial strains and growth conditions

ELW1 was typically grown on IB in batch culture in previously autoclaved 700 mL glass medium bottles sealed with screw caps and butyl rubber septa (Wheaton Scientific, Millville, NJ). The bottles contained a mineral salt medium (MSM) (100 mL) described by (17) and were inoculated to an initial optical density (OD_600_) of <0.02 using cells previously grown on agar plates of MV media under IB gas. IB (10% v/v gas phase) was added to the inoculated, sealed bottles using sterile plastic syringes fitted with sterile 0.1 µm pore-size disposable filters (Millipore Co., Bedford, MA). The flasks were then incubated in the dark at 30℃ for seven days in an environmental shaker operated at 150 rpm. Cells of *E. coli* TOP10 and DE3 were grown overnight in baffled Erlenmeyer flasks (50 mL) containing Luria-Bertani broth (5 mL) incubated in an environmental shaker operated at 30℃ and 150 rpm. Growth of all strains was determined by measuring optical density at 600 nm (OD_600_) using a WPA CO8000 Cell Density Meter (WPA CO8000, Biochrom, Cambridge, U.K.).

### Cloning *ibcK* of *Mycolicibacterium* sp. ELW1

The full annotated genome sequence of ELW1 was obtained through NCBI (Accession numbers CP032155.1 and CP032156.1). Gene clusters of interest were viewed in CLC Genomics Workbench, version 9.5 (Qiagen), and individually submitted to BLAST, where annotation of the gene and/or protein was confirmed. The *ibcK* gene encoding the putative epoxide hydrolase was amplified by PCR from colonies of ELW1 using iProof Taq (Bio-Rad, Hercules, CA), as described by the manufacturer using the forward primer 5′-GGG AAT TCC ATA TGA CAA CCG CCT CTT CCT TTG-3′ and the reverse primer 5′-CCC AAG CTT TCA TTT GAA AGC GGC CGT-3′. The reactions were conducted using a BioRad C1000 Touch Thermocycler with an initial denaturation step at 95℃ for 5 min followed by 30 cycles at 95℃ for 30 s, 67℃ for 30 s, and 72℃ for 105 s. A final extension was included at 72℃ for 5 min before holding at 12℃. PCR products were purified using a QIAprep Spin Miniprep Kit (Qiagen, Germantown, MD) and quantified using a NanoDrop spectrophotometer (Thermo Scientific, Wilmington, DE). The PCR product was ligated into *Eco*RV-digested pCR-Script using T4 ligase and used to transform TOP10 *E. coli. ibcK* was excised by digestion with *Nde*I and *Hind*III and ligated to similarly digested pET28a+ (Addgene, Watertown, MA), containing a 6×-His tag and a T7 promoter. The newly created pET28a-*ibcK* was used to transform TOP10 cells for future experiments as well as into BL21(DE3) cells for expression and protein purification. Colonies were screened for the presence of *ibcK* by PCR, and the sequence of *ibcK* in positive clones was confirmed using Sanger sequencing (Eton Bio; Research Triangle Park, NC).

### Induction and purification of IbcK

Cultures of BL21(DE3)*/*pET-28a-*ibcK* were grown overnight in LB medium containing kanamycin (50 µg/mL) and then diluted 1,000-fold into the same medium. After growth of these secondary cultures to an OD_600_ of ~0.6, expression of IbcK was induced by IPTG (100 µM) added from a freshly prepared aqueous stock solution (2 mM). After induction for 3 h, the cells were harvested by centrifugation (5000 × *g,* 5 min, 4℃). The resulting cell pellet was resuspended in IMAC buffer A (50 mM sodium phosphate, pH 7.4, containing 300 mM NaCl and 30 mM imidazole) (20 mL). The cells were then disrupted by three passages through a French press (FA-078, American Instrument Company, Silver Springs, MD) operated at ~3000 psi. The cell lysate was then centrifuged (5000 × *g,* 30 min, 4℃), and the resulting supernatant was filtered using a 0.45 µm syringe filter (Foxx Life Sciences, Salem, NH). The filtrate (22.5 mL) was then loaded onto a HisTrap HP (GE Healthcare, Uppsala, Sweden) column (5 mL) and eluted with IMAC buffer B (IMAC buffer A containing 300 mM imidazole) for a total of 15 column volumes. Fractions containing the appropriately sized protein, as determined by SDS-PAGE, were collected and pooled. These combined fractions were then dialyzed into buffer (10 mM Tris-HCl, pH 7.0 containing 150 mM NaCl) using a 20 kDa molecular weight cutoff Mini Slide-A-Lyzer (Thermo Fisher, Rockford, IL), following the manufacturer’s protocol. After dialysis, the protein solution was subjected to size exclusion chromatography using a HiPrep 16/60 Sephacryl S-200 column (GE Healthcare, Uppsala, Sweden). Collected fractions were analyzed by SDS-PAGE, and those fractions that contained the appropriately sized protein were combined and then dialyzed overnight into buffer (100 mM Tris, pH 7.5). The resulting dialyzed protein was quantified using a Pierce BCA Assay (Thermo Scientific, Rockford, IL) following the manufacturer’s protocol and then read using a POLARstar Galaxy plate reader (BMG Labtech, Offenburg, Germany) and stored in aliquots at −80℃.

### Transformation of 2-methyl-1,2-epoxypropane and other epoxides

The transformation of 2-methyl-1,2-epoxypropane (IBO) and other epoxides was examined in reactions conducted in glass serum vials (10 mL) sealed with butyl rubber stoppers and aluminum crimp seals (Wheaton Scientific, Millville, NJ). The vials contained buffer (~1 mL of 100 mM Tris-HCl, pH 7.5), and individual epoxides were added from freshly prepared stock solutions (100 mM) in buffer to give an initial concentration of 2 mM. The reaction vials were then preincubated for 5 min in a shaking water bath operated at 30℃ and 150 rpm. The reactions were initiated by the addition of purified IbcK (3–25 µg) to give a final reaction volume of 1 mL. The reaction vials were then returned to the shaking water bath, and aqueous samples (2 µL) were removed at the indicated times to determine the concentrations of residual epoxides by gas chromatography.

### Gas chromatography

The concentrations of epoxides in all reactions were determined by gas chromatography using a Shimadzu GC-14A gas chromatograph equipped with flame ionization detector and a stainless-steel column (0.3 × 60 cm) packed with Porapak Q 80-100 mesh (Grace Davison Discovery Science, Deerfield, IL). In all cases, the injection and detection temperatures were 200℃ and 220℃, respectively, and N_2_ was used as a carrier gas at 15 mL min^−1^. For the analysis of 1,2-epoxybutane, 1,2-epoxy-2-methylpropane, *cis*-2,3-epoxybutane, and *trans*-2,3-epoxybutane, the column was operated at 120℃. For the analysis of 1,2-epoxypentane and 2-(chloromethyl)oxirane (epichlorohydrin), the column was operated at 150℃. For the analysis of cyclohexene oxide, the column was operated at 170℃, while for the analysis of styrene oxide and 2-methyl-1,2-propanediol, the column was operated at 180℃. The gas chromatographs were interfaced to a Hewlett-Packard HP3395 integrator (Palo Alto, CA) for data collection and analysis.

### Crystallization and structure determination of IbcK

Purified IbcK was concentrated to 7 mg/mL using a 10 kDa molecular weight cutoff centrifugal concentrator (Vivaspin 20, GE Healthcare, Chicago, IL). Initial crystallization screens of 96 MCGS-1T conditions (Microlytic, Burlington, MA) were set up with the Phoenix Crystallization Robot (Art Robbins, Sunnyvale, CA) with 0.5 µL drops in a 1:1 well-to-drop ratio. Crystals grew in conditions MCGS1-53 (0.1 M MES:NaOH, pH 6.5, 1.6 M MgSO_4_) and MCGS1-95 (0.2 M NaCl, 0.1 M HEPES:NaOH, pH 7.5, 25% PEG 3350), although the MCGS1-53 crystals diffracted to higher resolution and were pursued for structure determination.

Crystal growth was observed after one month. Crystals were frozen in glycerol with the mother liquor and then shipped to the SER-CAT beamline at the Advanced Photon Source (SER-CAT, Atlanta, Georgia) for data collection at the ID beam. Data collection and refinement statistics are listed in Table S2. The data were scaled using HKL2000 (35). Initial phases were determined by molecular replacement with Phaser (36) implemented in the Phenix software package (37). The initial search model was derived from the epoxide hydrolase from *A. radiobacter* AD1 (PDB 1EHY) (31). The model was improved through alternate rounds of refinement with Phenix and manual rebuilding with Coot (38). Non-crystallographic symmetry did not reduce the R-factor or R-free, indicating that the monomers were sufficiently resolved independently. RMSD was calculated using the Matchmaker routine in Chimera (39). Figures were generated using Chimera (39) or Pymol (The PyMOL Molecular Graphics System, Version 2.57 Schrödinger, LLC). The structure is deposited in the Protein Data Bank as 9C2G.

### Size exclusion chromatography

The IbcK dimer was analyzed in solution by size exclusion chromatography with a Biosep-SEC S3000 column Phenomenex (Phenomenex, Torrance, CA, USA) with dimensions of 300 mm × 4.6 mm and a 5 μm particle size using an Acquity Ultra Performance Liquid Chromatography (UPLC) System (Waters Corporation, Milford, MA, USA). An isocratic elution was carried out with a mobile phase of 10 mM Tris-HCl, pH 7, containing 150 mM NaCl at a flow rate of 0.3 mL/min. 10 µL of sample was injected, and detection was performed at 220 nm and 280 nm using a tunable ultraviolet detector (TUV).

## SUPPLEMENTAL MATERIAL

Supplemental material aem.00393-25-s0001.pdf Figures S1 to S8; Tables S1 and S2.

ASM does not own the copyrights to Supplemental Material that may be linked to, or accessed through, an article. The authors have granted ASM a non-exclusive, world-wide license to publish the Supplemental Material files. Please contact the corresponding author directly for reuse.
