# Supplementary material for: Characterization of an isobutylene epoxide hydrolase (IbcK) from the isobutylene-catabolizing bacterium Mycolicibacterium sp. ELW1
Source: Appl Environ Microbiol. 2025 Aug 26;91(9):e00393-25. doi: 10.1128/aem.00393-25 (PMC12442371; doi:10.1128/aem.00393-25)
Supplement: Supplemental material — Figures S1 to S8; Tables S1 and S2. [file aem.00393-25-s0001.pdf]

Supplemental Material

Characterization of an Isobutylene Epoxide Hydrolase (IbcK) from the Isobutylene-Catabolizing Bacterium, *Mycolicibacterium* sp. ELW1

Authors: Nicholas W. Faulkner[1], John B. Joyce[1], Christy Smith[1], Paul Swartz[2], Robert B. Rose[2], Eric S. Miller[1,#] and Michael R. Hyman[1,#]

Address:
[1]Department of Plant and Microbial Biology, North Carolina State University, Raleigh, North Carolina 27695-7615, USA

[2]Department of Molecular and Structural Biochemistry, North Carolina State University, Raleigh, North Carolina 27695, USA

[#]Corresponding Authors: eric_miller@ncsu.edu; michael_hyman@ncsu.edu

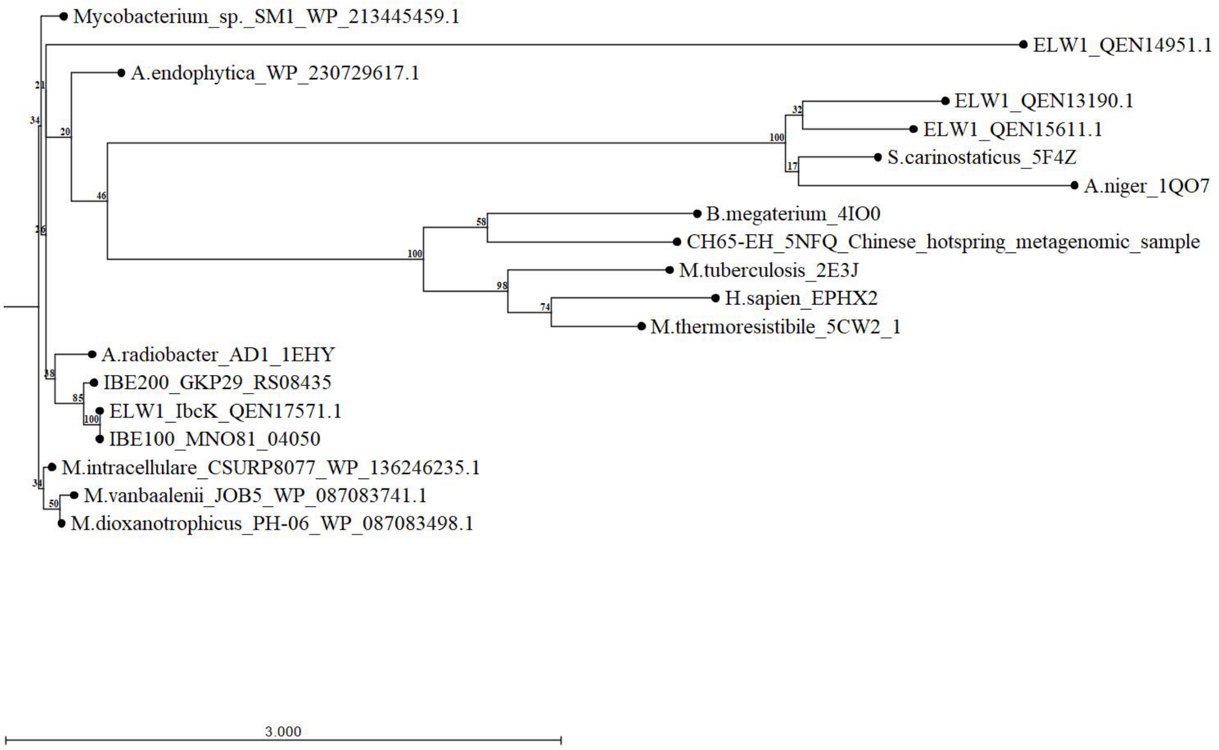

Figure S1: Evolutionary relationships of selected epoxide hydrolases.

Epoxide hydrolase protein sequences were aligned using the Classical Sequence Analysis (create alignment) tool using default settings in Qiagen CLC Genomics Workbench, version 25.0 (https://digitalinsights.qiagen.com). A phylogenetic tree was then created (CLC Maximum Likelihood Phylogeny method) from the aligned sequences that used the Neighbor-Joining method; the protein substitution model utilized WAG. Rate variation was included with default settings and Estimate Topology was selected. Bootstrapping analysis was performed with 100 replicates. The Tree is drawn to scale with branch lengths in the same units as the evolutionary distance units used to infer the phylogenetic tree. Bootstrapping value (%) are indicated on the branches and database accessions are given for each protein.

Figure S2. Expression vector pET28a-*ibcK*.

a. DNA was amplified from *Mycolicibacterium* sp. ELW1 cells using primers targeting *ibcK* (locus tag D3H54_RS30270; bp 69,001-69,912) on pELW1-1 (CP032156.1). An iProof Taq polymerase (BioRad) amplicon using forward primer 5'-GGG AAT TCC ATA TGA CAA CCG CCT CTT CCT TTG-3' and the reverse primer 5'-CCC AAG CTT TCA TTT GAA AGC GGC CGT-3' was purified and cloned into *Eco*RV-digested pCR-Script (Stratagene), transformed into *E. coli* TOP10 cells and the plasmid purified.

b. The pCR-Script:*ibcK* clone and pET28a(+) (Addgene) expression vector were digested with *Nde*I and *Hind*III, the *ibcK* fragment purified and the DNAs were ligated to yield pET28a-*ibcK*. The expression plasmid was transferred to *E. coli* BL21(DE3) for induction and purification of IbcK.

c. The wildtype sequence of *ibcK* in the pET28a-*ibcK* expression vector was confirmed by Sanger sequencing using universal primers "T7 Promoter" and "T7 Terminator" provided by Eton Bio (RTP, NC).

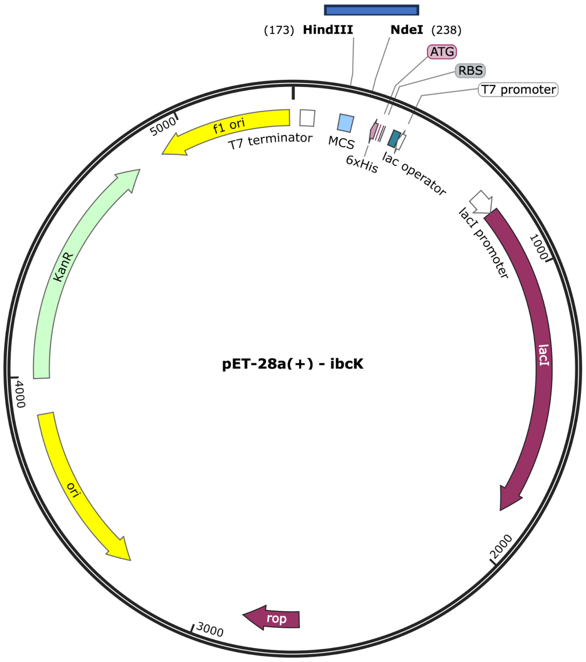

Table S1. Purification of the recombinant *Mycolicibacterium* sp. ELW1 IbcK protein from *E. coli*/pET28a-*ibcK* cells.

Step	Total Protein (mg)	Total Units[a]	Specific Activity[b]	Yield (%)	Purification (fold)
Whole Cells	2100	3965	1.9	100	1
Cell Extract	206.1	783	3.8	19.7	2
Ni-NTA	20.6	601	29.1	15.2	15.4
Size Exclusion	19.5	413	21.2	10.4	11.2

a, Units are in µmoles of substrate consumed per minute.

b, Specific activity units are µmoles consumed min^{-1} mg $protein^{-1}$.

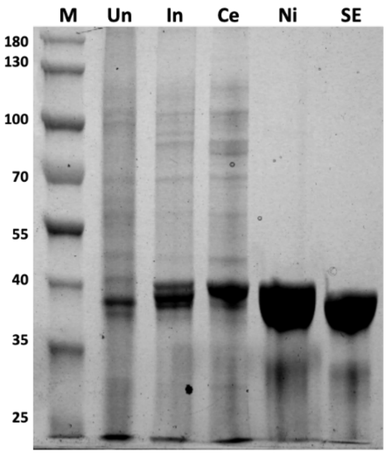

Figure S3a. SDS-PAGE image of IbcK EPH induction and purification. Equal amounts of total protein were loaded and then stained with Coomassie Blue R. Pre-stained PAGERuler (Thermo Scientific) was used as mass markers. Samples are as described for Table S1: M, mass markers; Un, uninduced cells; In, induced cells; Ce, induced cell extract; Ni, HisTRAP nickel column elution; SE, size-exclusion column fraction.

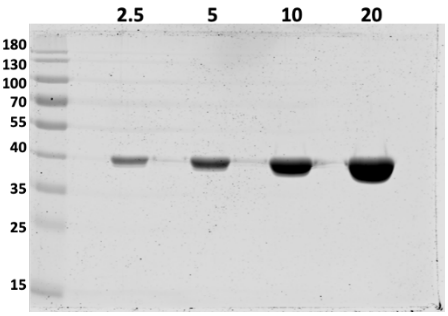

Figure S3b. SDS-PAGE image of purified IbcK EPH. A concentration gradient of the purified enzyme (Figure S2a), 2.5 to 20 ug, used in the assays and structure analysis of this report. Mass standards are as noted on the left, 15 kDa – 180 kDa. The mass of IbcK+6His predicted from the sequence is 36.8 kDa.

Table S2. Crystallographic statistics of the IbcK structure.

PDB entry	9C2G
space group	$P2_12_12_1$
wavelength (Å)	1
unit cell (Å)	48.417, 181.005, 221.720<br>90.00, 90.00, 90.00
resolution range (Å)	140.21-2.29 (2.35-2.29)
no. unique reflections	89143(4910)
completeness (%)	91.4 (78.6)
Wilson B-factor (Å^2)	37.98
mean I/sI	5.8
**Refinement**	
resolution range (Å)	47.30-2.29 (2.35-2.29)
R_{work}(%)	17.76 (22.47)
R_{free} (%)	22.72 (29.22)
total non-hydrogen atoms	14480
macromolecules	14100
ligands	104
waters	276
no. of protein residues	1757
rmsd Bonds (Å)	0.003
rmsd Angles (Å)	0.567
Ramachandran favored (%)	96.16
Ramachandran outliers (%)	0.29
Average B-factor (Å^2)	
macromolecules	45.76
solvent	38.87

Figure S4. Crystal packing of IbcK in the asymmetric unit. The asymmetric unit contains six monomers of IbcK epoxide hydrolase with each monomer colored differently. There are two dimers (chains E and F, yellow-red and chains A and B blue-turquoise) and two monomers (chains C and D, green) that form dimers with symmetry-related monomers. Refinement statistics for the model are given in Table S2.

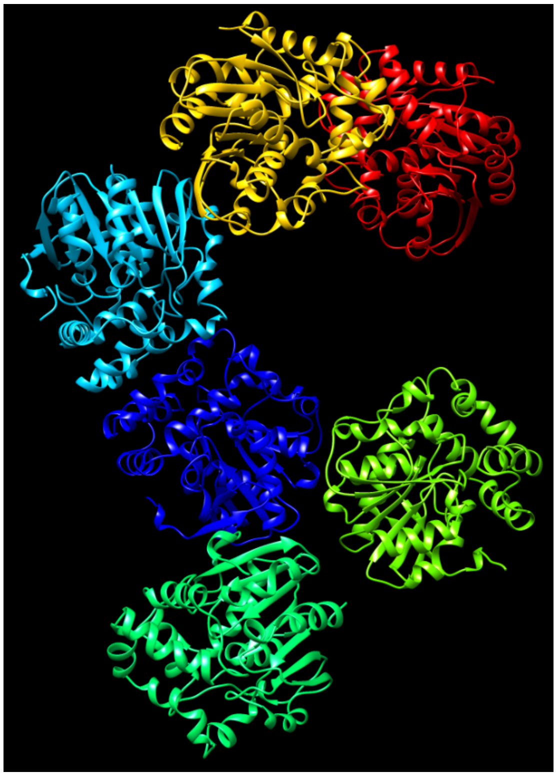

Figure S5. Size exclusion chromatography of purified IbcK. A) Two peaks eluted from the size exclusion column (black line), compared with gel filtration standards (gray line) (Biorad #1511901). **B)** The molecular weights of the peaks were calculated by plotting the $\log_{10}$(MW) vs elution time for the standards (open circles) fit to a linear trendline (dotted line, Microsoft Excel, Version 16.87). The MW of the IbcK peaks were determined from the elution times (cross marks) from the equation of the trend line. The calculated MWs were: Peak 1, 67,200 Da, Peak 2, 46,200 Da. The calculated MW of an IbcK monomer is 36,789 Da. This suggests an equilibrium exists between monomer and dimer in solution. The plot is based on the equation: (Ve – Vo)/(Vc – Vo)= a $\log_{10}$(MW) + b with Ve the elution volume, Vo the void volume, Vc the column volume.

A B

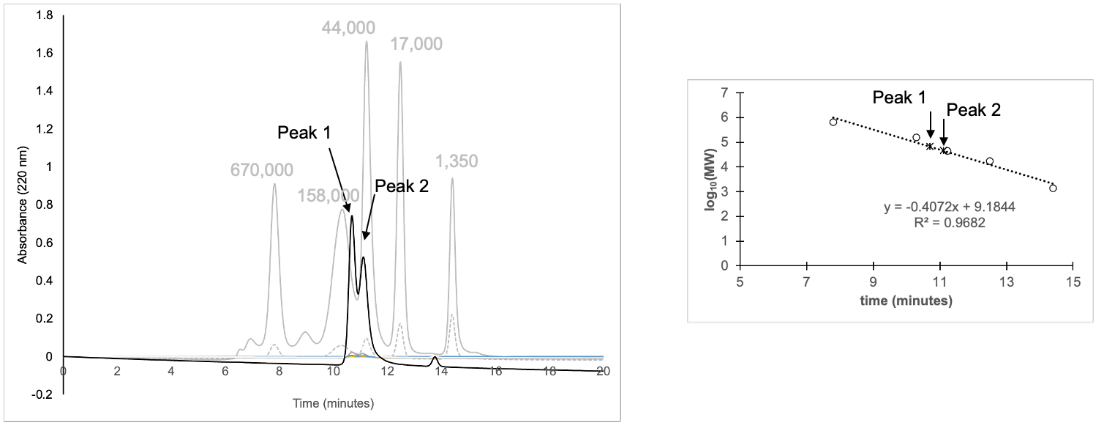

Figure S6. Alignment of seven bacterial epoxide hydrolase sequences. The conserved active site residues are labeled. The numbering is for the IbcK structure. (Structural alignment with the Chimera Matchmaker algorithm (1). Displayed with ESPript 3.0 (2). 9C2G – IbcK (current paper), 4IO0 – Bm - *Bacillus megaterium* (3), 5NG7 – CH65 metagenomic sequence from Chinese hot springs (4), 3G02 – An – *Aspergillus niger* (5), 1EHY – AgAD1 – *Agrobacterium radiobacter* AD1 (6), 5F4z – Sc - *Streptomyces carzinostaticus* (unpublished), 2E3J – Mt – *Mycobacterium tuberculosis* (7).

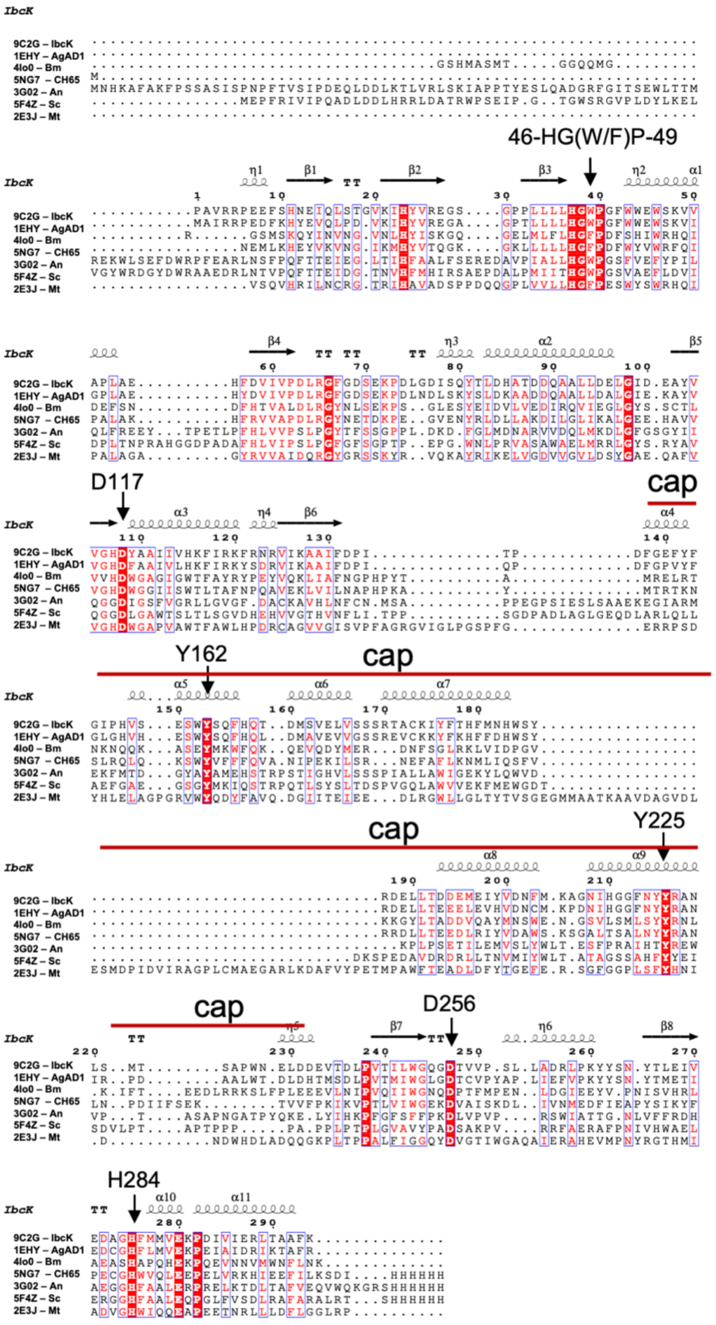

Figure S7. Other microbial epoxide hydrolases utilize the cap domain as a dimer interface. Not all epoxide hydrolase dimers use this interface. E) shows an example of a dimer that utilizes a different interface. The interfaces are colored orange and dark blue showing residues within 5.0 Å of the other monomer (green and cyan).

A) IbcK dimerization interface (this paper).

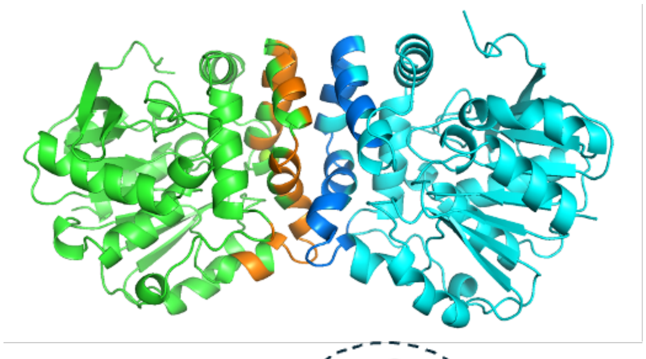

B) *Streptomyces carzinostaticus* enzyme (pdb 5f4Z). The dimer interface consists of the cap domain helices plus an additional loop, part of the N-terminal helical sequence (gray, enclosed in dotted line; unpublished).

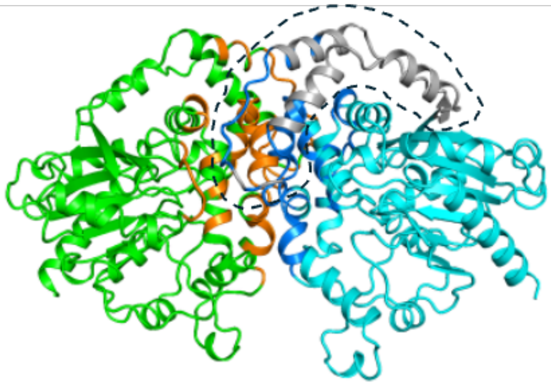

C) *Aspergillus niger* enzyme (3G02). The dimer interface is like pdb 5f4Z above (5).

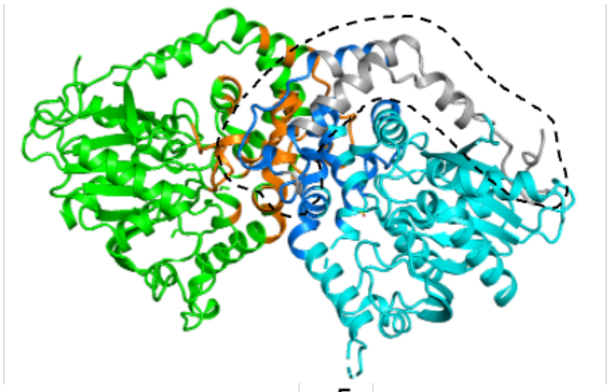

D) Thermophilic enzyme from a metagenome derived from a hot spring (pdb 5NG7). The interface between cap domains differs from IbcK with a longer helix $\alpha5$ and helix $\alpha9$ (4).

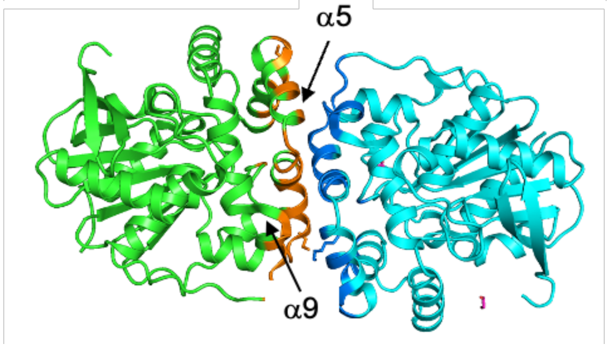

E) Other dimer interfaces are possible. The *M. tuberculosis* dimer (pdb 2E3J) (cyan and green) interface spans the core and cap domains. The position of the IbcK dimer is shown in grey superimposed on one monomer of the *M. tuberculosis* dimer indicating the position of the cap interface (7).

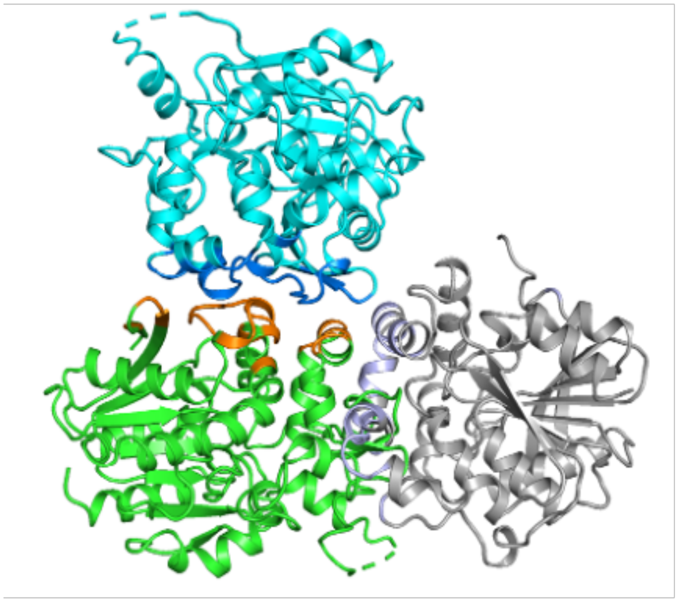

Figure S8. Glycerol in the active site of IbcK (sticks) is shown through a narrow channel from the surface of a monomer in the IbcK dimer structure. The surface representation of the IbcK monomer (chain A) is colored as a Coulombic surface (coloring: red: -10kcal/mole, white neutral, blue: +10 kcal/mole). The surface around the channel is mostly hydrophobic, but the surface overall is negatively charged. The unknown molecule blocking this channel in our structure (modeled as a poly-carbon chain) is not shown in this figure. The IbcK monomer (9C2G) is oriented with the cap facing up and tilted to the back of the page (dotted line). The figure was drawn with Chimera and colored using the Coulombic Surface algorithm in Chimera (8).

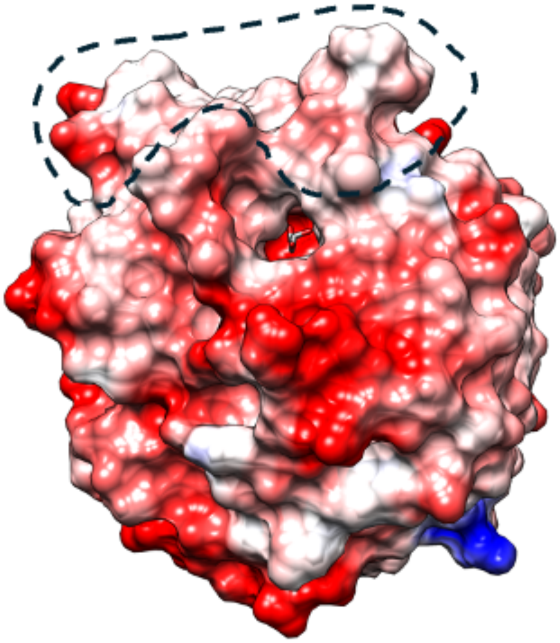

Supplemental References

1. Meng EC, Pettersen EF, Couch GS, Huang CC, Ferrin TE. 2006. Tools for integrated sequence-structure analysis with UCSF Chimera. BMC Bioinformatics 7:339.

2. Robert X, Gouet P. 2014. Deciphering key features in protein structures with the new ENDscript server. Nucleic Acids Res 42:W320-324.

3. Kong X-D, Yuan S, Li L, Chen S, Xu J-H, Zhou J. 2014. Engineering of an epoxide hydrolase for efficient bioresolution of bulky pharmaco substrates. Proc Natl Acad Sci 111:15717–15722.

4. Ferrandi EE, Sayer C, De Rose SA, Guazzelli E, Marchesi C, Saneei V, Isupov MN, Littlechild JA, Monti D. 2018. New thermophilic α/β class epoxide hydrolases found in metagenomes from hot environments. Front Bioeng Biotechnol 6:144.

5. Reetz MT, Bocola M, Wang L-W, Sanchis J, Cronin A, Arand M, Zou J, Archelas A, Bottalla A-L, Naworyta A, Mowbray SL. 2009. Directed evolution of an enantioselective epoxide hydrolase: uncovering the source of enantioselectivity at each evolutionary stage. J Am Chem Soc 131:7334–7343.

6. Nardini M, Ridder IS, Rozeboom HJ, Kalk KH, Rink R, Janssen DB, Dijkstra BW. 1999. The x-ray structure of epoxide hydrolase from *Agrobacterium radiobacter* AD1. An enzyme to detoxify harmful epoxides. J Biol Chem 274:14579–14586.

7. Biswal BK, Morisseau C, Garen G, Cherney MM, Garen C, Niu C, Hammock BD, James MNG. 2008. The Molecular structure of epoxide hydrolase B from *Mycobacterium tuberculosis* and its complex with a urea-based inhibitor. J Mol Biol 381:897–912.

8. Pettersen EF, Goddard TD, Huang CC, Couch GS, Greenblatt DM, Meng EC, Ferrin TE. 2004. UCSF Chimera--a visualization system for exploratory research and analysis. J Comput Chem 25:1605–1612.
